# Puerarin attenuates intracerebral hemorrhage‐induced early brain injury possibly by PI3K/Akt signal activation‐mediated suppression of NF‐κB pathway

**DOI:** 10.1111/jcmm.16679

**Published:** 2021-06-27

**Authors:** Jun Zeng, Shizhong Zheng, Yizhao Chen, Yaoming Qu, Jiayu Xie, Enhui Hong, Hongzhu Lv, Rui Ding, Liang Feng, Zhichong Xie

**Affiliations:** ^1^ Department of Neurosurgery Zhujiang Hospital The Engineering Technology Research Center of Education Ministry of China The National Key Clinical Specialty The Neurosurgery Institute of Guangdong Province Guangdong Provincial Key Laboratory on Brain Function Repair and Regeneration Southern Medical University Guangzhou China; ^2^ Department of Neurosurgery Huashan Hospital Institute of Neurosurgery Shanghai Medical College Fudan University Shanghai China; ^3^ Department of Neurosurgery The Seventh Affiliated Hospital of Sun Yat‐Sen University Shenzhen China; ^4^ Department of Neurosurgery The First Affiliated Hospital of Guangzhou Medical University Guangzhou China; ^5^ Department of Radiology Zhujiang Hospital Southern Medical University Guangzhou China; ^6^ Department of Neurosurgery The Third Affiliated Hospital of Sun Yat‐Sen University Guangzhou China; ^7^ Department of Neurosurgery Chenzhou No. 1 People's Hospital Chenzhou China

**Keywords:** ICH, EBI, puerarin, NF‐κB, PI3K

## Abstract

Intracerebral hemorrhage (ICH) can induce intensively oxidative stress, neuroinflammation, and brain cell apoptosis. However, currently, there is no highly effective treatment available. Puerarin (PUE) possesses excellent neuroprotective effects by suppressing the NF‐κB pathway and activating the PI3K/Akt signal, but its role and related mechanisms in ICH‐induced early brain injury (EBI) remain unclear. In this study, we intended to observe the effects of PUE and molecular mechanisms on ICH‐induced EBI. ICH was induced in rats by collagenase IV injection. PUE was intraperitoneally administrated alone or with simultaneously intracerebroventricular injection of LY294002 (a specific inhibitor of the PI3K/Akt signal). Neurological deficiency, histological impairment, brain edema, hematoma volume, blood–brain barrier destruction, and brain cell apoptosis were evaluated. Western blot, immunohistochemistry staining, reactive oxygen species (ROS) measurement, and enzyme‐linked immunosorbent assay were performed. PUE administration at 50 mg/kg and 100 mg/kg could significantly reduce ICH‐induced neurological deficits and EBI. Moreover, PUE could notably restrain ICH‐induced upregulation of the NF‐κB pathway, pro‐inflammatory cytokines, ROS level, and apoptotic pathway and activate the PI3K/Akt signal. However, LY294002 delivery could efficaciously weaken these neuroprotective effects of PUE. Overall, PUE could attenuate ICH‐induced behavioral defects and EBI possibly by PI3K/Akt signal stimulation‐mediated inhibition of the NF‐κB pathway, and this made PUE a potential candidate as a promising therapeutic option for ICH‐induced EBI.

## 1 INTRODUCTION

Intracerebral hemorrhage (ICH) belongs to the most lethal subtype of stroke^1^ and accounts for 10‐15% of all strokes in the USA, Europe, and Australia and for Asia, up to 20‐30%.^1^, ^2^ ICH can lead to high mortality and morbidity risks, and only 20% of sufferers can realize functional independence at 6 months following ICH.^1^, ^2^, ^3^ Although considerable progress has been achieved, there remains no acknowledged treatment available at present.^4^ Neuroinflammation and oxidative stress (OS) play crucial roles in the pathological process of ICH‐induced early brain injury (EBI), and efficient blockage might be a potential strategy for ICH therapy.^1^, ^2^, ^4^, ^5^, ^6^

Puerarin (PUE), as a primary bioactive compound of *Pueraria* *lobata* roots,^7^, ^8^ has exhibited potently neuroprotective activity in multiple central nervous system (CNS) disorders, including stroke,^9^, ^10^, ^11^, ^12^, ^13^, ^14^, ^15^, ^16^ subarachnoid hemorrhage (SAH),^17^ traumatic brain injury (TBI),^18^ spinal cord injury (SCI),^19^, ^20^, ^21^, ^22^, ^23^ Alzheimer's disease (AD),^24^, ^25^, ^26^, ^27^, ^28^ and Parkinson's disease (PD).^29^, ^30^, ^31^, ^32^, ^33^ Research studies have shown that PUE can generate brain‐protective effects by activating the phosphatidylinositol 3‐kinase (PI3K)/Akt signal against acute CNS disorders^18^, ^19^, ^34^ and neurodegenerative diseases.^27^, ^28^, ^29^, ^35^ Besides, PUE also could exert beneficial effects by repressing nuclear factor‐κB (NF‐κB) pathway activation and pro‐inflammatory cytokine production [tumor necrosis factor‐α (TNF‐α), interleukin‐6 (IL‐6), and interleukin‐1β (IL‐1β)].^11^, ^12^, ^23^ Moreover, several reports have suggested that stimulation of the PI3K/Akt signal can restrain the activation of the NF‐κB pathway,^36^ and LY294002 (a specific inhibitor of PI3K/Akt signal) can reverse this effect.^36^, ^37^, ^38^ Furthermore, activation of the PI3K/Akt signal can relieve ICH‐induced neurobehavioral deficiency and EBI,^39^, ^40^, ^41^, ^42^, ^43^ and stimulation of the NF‐κB signal pathway plays a highly detrimental role in EBI after ICH, and its inhibition can produce notable neuroprotective effects.^44^, ^45^ However, it remains unclear whether PUE holds similar brain‐protective effects in ICH‐induced EBI and related mechanisms.

## 2 MATERIALS AND METHODS

### 2.1 Animals

Male adult rats (Sprague–Dawley, 280‐320 g) were purchased from the Animal Experiment Center of Southern Medical University (Guangzhou, Guangdong, China). The research protocol and animal care were authorized by the Southern Medical University Ethics Committee and were performed according to the National Institutes of Health guidelines on care and use of animals.

### 2.2 Experimental design and group

In our research, three experiments were designed and orderly performed. The specific experimental design and group were detailed in *Supplementary material* *s*, respectively (Fig. S1, Fig. S2, Fig. S3).

### 2.3 Rat ICH model

ICH was induced in rats as mentioned in previous reports.^44^, ^46^, ^47^ Simply, after anesthetized with pentobarbital sodium (45 mg/kg) (Cat. No.: P3761, Sigma‐Aldrich, St. Louis, MO, USA), a middle scalp incision was made in rats to expose the bregma. Next, a burr hole (1 mm in diameter) was drilled on the right skull, and a microsyringe (5 μl) was directly inserted into the right striatum (bregma: lateral 3.5 mm, anterior 0.1 mm, and ventral 6.0 mm). Collagenase IV (1 μl, 0.2 U/μl in 0.9% normal saline) (Cat. No.: C1889, Sigma‐Aldrich, St. Louis, MO, USA) was injected after 10 min. After in situ for another 10 min, the syringe was slowly moved out. Sham group rats were dealt with an identical method, except that only 1 μl of 0.9% normal saline was injected. The rats were then placed in separate cages and were provided with standard food and water.

### 2.4 Drug delivery

Dimethyl sulfoxide (DMSO) (Cat. No.: D5879, Sigma‐Aldrich, St. Louis, MO, USA) solution of PUE (Cat. No.: P5555, Sigma‐Aldrich, St. Louis, MO, USA) (100 mg/ml) was prepared. The rats were injected intraperitoneally with either PUE at 50 mg/kg and 100 mg/kg, or just DMSO solution 30 min before modeling and at the time points of 30 min, 6 h, 12 h, 24 h, and 48 h after ICH. We selected the dosages and administration route based on previous studies, and intraperitoneal injection of PUE at 50 mg/kg and 100 mg/kg could both produce significantly neuroprotective effects.^11^, ^14^, ^17^, ^22^ LY294002 (Cat. No.: HY‐10108, MedChemExpress, Monmouth Junction, NJ, USA) in 25% DMSO with phosphate‐buffered saline (PBS) was then intracerebroventricularly administrated (50 mmol/L, 10 μl; bregma: lateral 1.4 mm, posterior 0.8 mm, and ventral 3.6 mm) according to previous reports.^48^, ^49^

### 2.5 Behavioral deficiency

The modified neurological severity score (mNSS) scale was applied to estimate the neurobehavioral deficiency at 24 h and 72 h following ICH, which was executed by two experienced researchers who were blinded to animal groups.^44^, ^45^, ^50^, ^51^ The scale is composed of sensory, motor, reflex, and balance tests, and a higher score means more severe neurological injury.^44^, ^45^, ^50^, ^51^

### 2.6 Paraffin sections

Paraffin sections of rat brains were made, as previously described.^44^, ^45^, ^46^, ^52^ In brief, after transcardial perfusion with PBS followed by 4% paraformaldehyde, the rat brains were taken out and performed postfixation in the same fixation solution (4°C, 24 h). After being dehydrated and vitrified, the brain samples were embedded into paraffin. Then, after being dewaxed and rehydrated, the brain sections (4 μm thickness) were used to conduct hematoxylin and eosin (H&E), immunohistochemistry (IHC), and terminal deoxynucleotidyl transferase‐mediated biotinylated dUTP nick‐end labeling (TUNEL) staining.

### 2.7 H&E staining

H&E staining was performed as our previous method.^44^, ^46^ In brief, the prepared brain sections were immersed into eosin for 10 s and then into hematoxylin for 5 min. After dealing with graded ethanol and xylene, the brain sections were mounted and imaged with a microscope (DM2500, Leica, Germany).

### 2.8 Hematoma volume

Hematoma volume was assessed as previously reported with some modifications.^53^, ^54^ In brief, serially coronal sections (2 mm thickness) of rat brains were prepared and imaged with a digital camera. Then, the acquired images were utilized to calculate the hematoma volume with the following formula: *V *= *T*_1_*S_1_ + *T*_2_*S_2_ + … + *T*_n_*S_n_ [*V*: hematoma volume (mm^3^), *T*: slice thickness, *S*: hematoma area, *n*: serial number of brain slices].

### 2.9 Brain water content

Brain water content (BWC) was measured by the wet/dry weight method, as previously described.^44^, ^55^, ^56^ In brief, at 24 h or 72 h following ICH, the rat brains were harvested and separated into five parts (ipsilateral and contralateral cerebral cortices, ipsilateral and contralateral basal ganglia tissues, and cerebellum). After wet weight was obtained, the brain samples were dried to get dry weight (100°C, 24 h). BWC was calculated with the following formula: [(*M*_1_−*M*_2_)]/*M*_1_*100% (*M*_1_: wet weight, *M*_2_: dry weight).

### 2.10 Blood–brain barrier

Evans blue (EB) dye (Wako Pure Chemical Industries, Japan) was applied to assess the disruption of the blood–brain barrier (BBB), as reported previously.^44^, ^52^, ^57^ In brief, after intravenous injection of 2% EB for 2 h, the rats were transcardially perfused and then right brain hemispheres were isolated. Next, each brain sample was further immersed into 50% trichloroacetic acid. After being homogenized and centrifuged, the supernatant (1 ml) of each brain sample was collected and diluted with ethanol (1:3). Finally, the signal was acquired with a multifunctional microplate reader (excitation: 620 nm; emission: 680 nm; SpectraMax M5, Molecular Devices, USA). The extravasation of EB dyes was described in micrograms/gram brain tissue weight.

### 2.11 TUNEL staining

At 24 h and 72 h after ICH, the brain samples were obtained and paraffin sections were prepared. TUNEL staining was performed with an In Situ Cell Death Detection Kit (Cat. No.: 11684795910, Roche, Basel, Basel‐Stadt, Switzerland) as our previous method.^45^, ^46^ The stained sections were imaged with a fluorescent microscope (Nikon, Nikon Eclipse C1, Japan), and TUNEL^+^ cells were counted in a blinded manner.

### 2.12 IHC staining

IHC staining was performed as previously reported.^44^, ^45^, ^55^ In brief, antigen retrieval of the brain sections was executed through heat treatment for about 21 min in Tris–ethylenediaminetetraacetic acid solution (0.001 mol/L). After dealing with 0.3% H_2_O_2_ for 10 min and antigen blocking with 5% bovine serum albumin for 20 min, the samples underwent overnighted co‐incubation at 4°C with the following primary antibodies: cleaved caspase‐3 (Cat. No.: #9664, 1:800), ^Ser536^p‐NF‐κB p65 (Cat. No.: #3033, 1:200), NF‐κB p65 (Cat. No.: #8242, 1:200) [Cell Signaling Technology (CST), Danvers, MA, USA], 3‐nitrotyrosine (3‐NT) (Cat. No.: ab61392, 1:200, Abcam, Cambridge, UK), and 8‐hydroxyguanosine (8‐OHdG) (Cat. No.: ab48508, 1:200, Abcam). Then, the brain slices were orderly incubated with corresponding secondary antibodies and horseradish peroxidase–streptavidin for 20 min. After further reaction with 3, 3‐diaminobenzidine and being counterstained with hematoxylin, representative IHC images were obtained using a microscope (DM2500, Leica, Germany).

### 2.13 Western blot

Western blot (WB) was carried out as our previous method.^44^, ^45^, ^55^ The following primary antibodies were applied: ^Ser536^p‐NF‐κB p65 (Cat. No.: #3033), NF‐κB p65 (Cat. No.: #8242), ^Ser473^p‐Akt (Cat. No.: #4060), Akt (Cat. No.: #9272), Bax (Cat. No.: #2772), cleaved caspase‐3 (Cat. No.: #9664) (1:1000, CST, Danvers, MA, USA), Bcl‐2 (Cat. No.: ab59348, 1:500, Abcam), ^Tyr607^p‐PI3K (Cat. No.: ab182651, 1:1000, Abcam), and PI3K (Cat. No.: BSM‐33219 M, 1:1000, Bioss, Beijing, China). β‐actin (Cat. No.: ab8227, 1:1000, Abcam) and Lamin A (Cat. No.: ab26300, 1:3000, Abcam) were employed as the internal reference. WB protein bands were quantified by ImageJ software (National Institutes of Health, Baltimore, MD, USA). Protein expression levels were indicated by the ratio of interest protein bands to that of β‐actin or Lamin A bands.

### 2.14 Enzyme‐linked immunosorbent assay

At 24 h after ICH, the brain samples were collected and used to detect pro‐inflammatory cytokine levels using a Rat TNF‐α ELISA Kit (Cat. No.: SEA133Ra), Rat IL‐1β ELISA Kit (Cat. No.: SEA563Ra), and Rat IL‐6 ELISA Kit (Cat. No.: SEA079Ra) (Cloud‐Clone Corp, Houston, TX, USA) as our previous method.^44^ In brief, the prepared brain samples were put into related enzyme wells, which were pre‐coated with rat TNF‐α, IL‐6, or IL‐1β antibodies and then incubated for about 1.5 h at 37°C. After being washed thrice with PBS, the brain samples were further reacted with chromogen solutions A and B. Finally, the brain samples were detected at 450 nm by using a multifunctional microplate reader (SpectraMax M5, Molecular Devices, USA).

### 2.15 Measurement of reactive oxygen species

The reactive oxygen species (ROS) level was detected using a ROS assay kit (Cat. No.: E004, Nanjing Jiancheng Bioengineering Institute, Jiangsu, China) at 24 h after ICH.^44^ In brief, the prepared brain tissue samples were treated using 2,7‐dichlorofluorescein diacetate (DCFH‐DA) probes (60 μM, 37℃, 60 min), and after washing thrice with PBS, the samples were detected with a multifunctional microplate reader (excitation: 500 nm, emission: 525 nm; SpectraMax M5, Molecular Devices, USA).

### 2.16 Statistical analysis

All data were expressed as means ± standard deviation (SD). Data analyses were conducted with SPSS 19.0 (SPSS, Inc., Chicago, IL, USA), and related diagrams were prepared with GraphPad Prism 5 (GraphPad, Inc, San Diego, CA, USA). All data were analyzed using the Shapiro–Wilk and Levene tests. If data satisfy normal distribution and homogeneity of variance, one‐way analysis of variance (ANOVA) was considered, and then the least significant difference (LSD) test was applied to compare the difference among multiple experimental groups; conversely, for unsatisfied data, Dunnett's T3 test was adopted. The *P*‐value was statistically significant when <0.05.

## 3 RESULTS

### 3.1 PUE could alleviate ICH‐induced behavioral defects and histological injury

**FIGURE 1:**
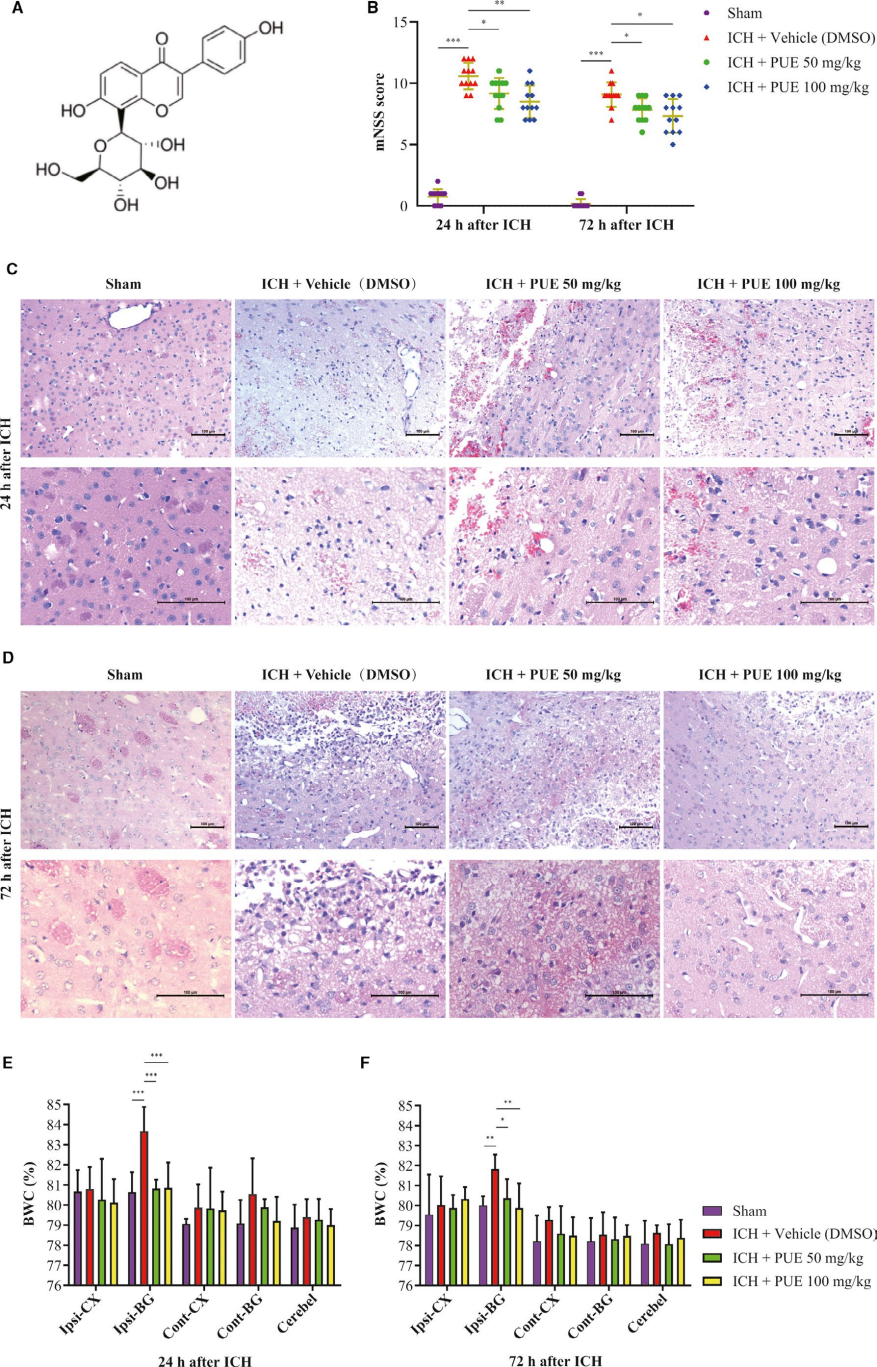
Chemical structure of PUE and effects of PUE treatment at doses of 50 and 100 mg/kg on ICH‐induced EBI. Chemical structure of PUE (A). At 24 h and 72 h after ICH, PUE administration (50 and 100 mg/kg) could significantly reduce the neurological deficits evaluated with an mNSS scale (B) (n = 12 rats/group; 24 h and 72 h: Dunnett's T3 test). Analogously, PUE delivery at both doses could markedly decrease the histological injury shown by H&E staining (C, D) (n = 6 rats/group) and BWC (E, F) (n = 6 rats/group; 24 h and 72 h: LSD test) measured with the dry wet weight method at 24 h and 72 h post‐ICH, respectively. Scale bar = 100 μm. Values are presented as means ± SD. ***P < .001; **P < .01; *P < .05

In our experiment, a total of 341 rats were used, and five rats died (5/341 rats, related mortality: approximately 1.5%). The schedule of research, including ICH induction of rats, treatments with different agents [especially for the time points of administration, such as PUE, LY294002, and vehicle (DMSO)], and corresponding experimental assessments, is shown (Fig. S4). The mNSS scores markedly increased at 24 h (*P* < .001) and 72 h (*P* < .001) (Figure 1B) after ICH. PUE delivery could notably reduce the mNSS scores at 50 mg/kg (*P* < .05, 24 h and 72 h) and 100 mg/kg (*P* < .01, 24 h; *P* < .05, 72 h), yet the beneficial effects of two treatment dosages were not significantly different (*P* > .05, 24 h and 72 h) (Figure 1B). Analogously, H&E staining also indicated that PUE could alleviate ICH‐induced histological injury at both 50 mg/kg and 100 mg/kg at 24 h (Figure 1C) and 72 h (Figure 1D) following ICH.

### 3.2 PUE could relieve ICH‐induced brain edema and BBB destruction

**FIGURE 2:**
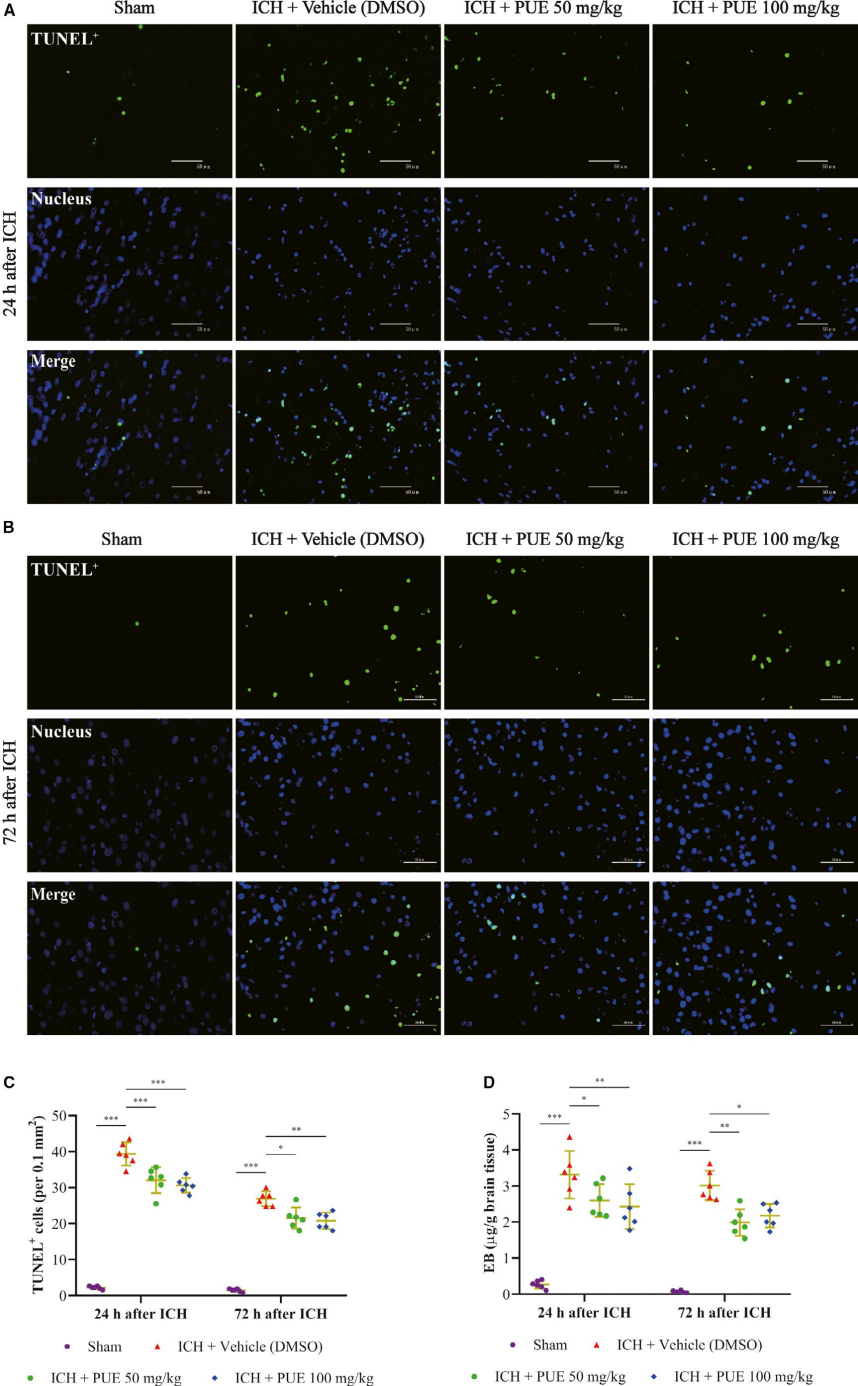
Effects of PUE treatment at doses of 50 and 100 mg/kg on the apoptosis level of brain cells and disruption of BBB. Typical microscopic images of TUNEL^+^ cells from the perihematomal brain tissue are shown (A, B), and relatively quantitative analyses of TUNEL^+^ cells (C) at 24 h and 72 h after ICH induction were obtained (n = 6 rats/group; 24 h: LSD test; 72 h: Dunnett's T3 test). The quantitative analyses of extravasated EB dyes were exhibited at 24 h and 72 h post‐ICH (D) (n = 6 rats/group; 24 h: LSD test; 72 h: Dunnett's T3 test). Scale bar = 50 μm. Values are presented as means ± SD. ***P < .001; **P < .01; *: *P* < .05

After ICH, BWC obviously increased (*P* < .001, 24 h; *P* < .01, 72 h) (Figure 1E,F). PUE at 50 mg/kg (*P* < .001, 24 h; *P* < .05, 72 h) and 100 mg/kg (*P* < .001, 24 h; *P* < .01, 72 h) could significantly reduce an ICH‐induced increase in BWC (Figure 1E,F). However, no obvious distinction for PUE’s beneficial effects was observed (*P* > .05, 24 h and 72 h) (Figure 1E,F). Extravasation of EB dyes clearly increased after ICH (*P* < .001, 24 h and 72 h) (Figure 2D), and PUE (50 mg/kg) could significantly reduce ICH‐induced leakage of EB dyes (*P* < .05, 24 h; *P* < .01, 72 h). Besides, PUE (100 mg/kg) also could notably repress ICH‐induced EB dye extravasation (*P* < .01, 24 h; *P* < .05, 72 h) (Figure 2D). Similarly, no significant difference was observed between 50 and 100 mg/kg for PUE treatment (*P* > .05, 24 h and 72 h) (Figure 2D).

### 3.3 PUE could drop ICH‐induced brain cell apoptosis and hematoma volume

**FIGURE 3:**
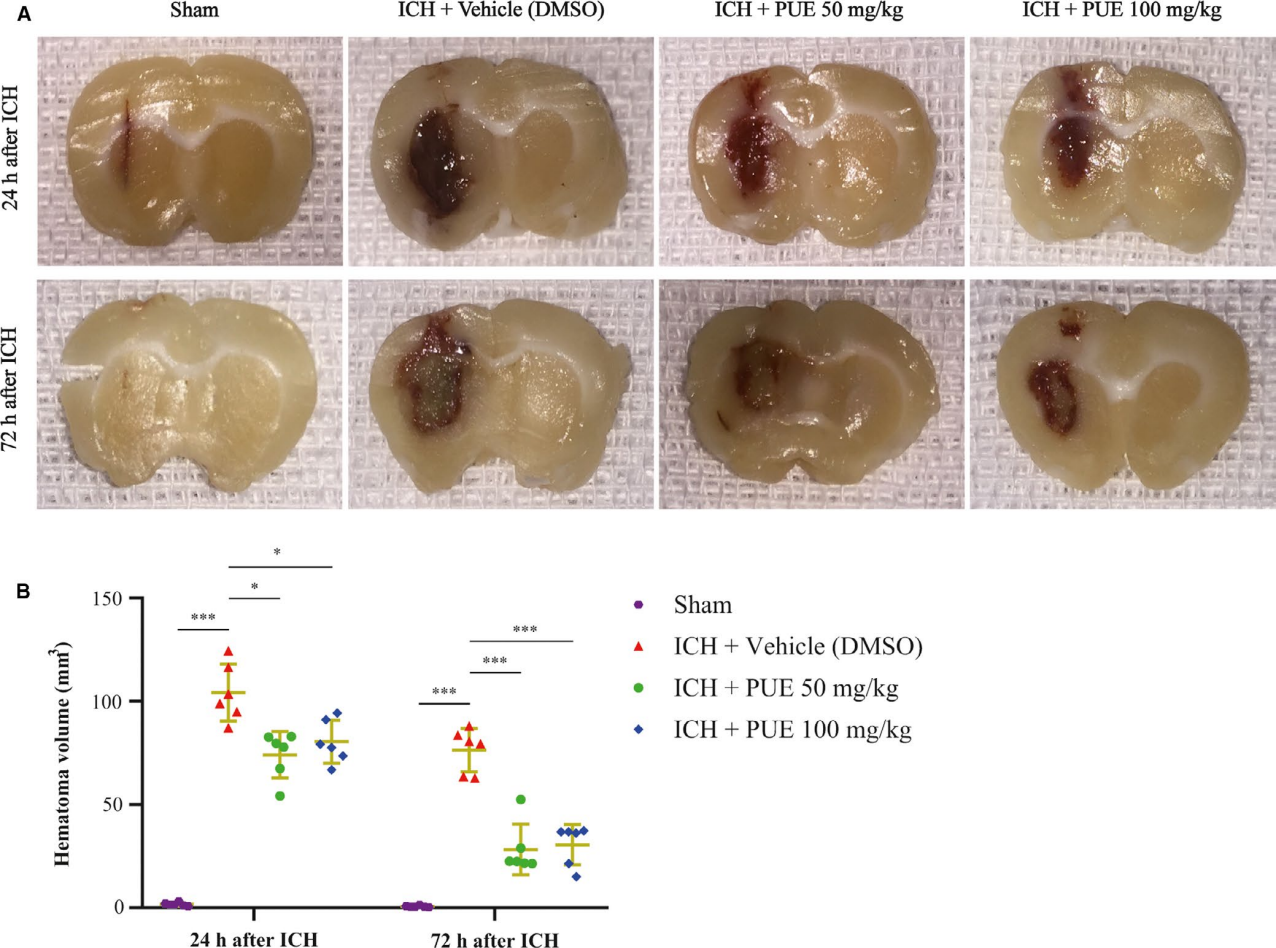
Effects of PUE treatment at doses of 50 and 100 mg/kg on the hematoma formation at 24 h and 72 h after ICH. At 24 h and 72 h after ICH induction, typical macroscopical images of rat brains were obtained by autopsy and exhibited (A). Relatively quantitative analyses of hematoma volume were carried out and are shown (B) (n = 6 rats/group; 24 h and 72 h: Dunnett's T3 test). Values are reported as means ± SD. ***P < .001; **P < .01; *P < .05

Typically, microscopic images of TUNEL staining were obtained at 24 h and 72 h after ICH (Figure 2A,B). Apoptotic brain cells significantly increased after ICH (*P* <.001, 24 h and 72 h) (Figure 2C). Treatment of PUE at 50 mg/kg (*P* < .001, 24 h; *P* < .05, 72 h) and 100 mg/kg (*P* < .001, 24 h; *P* < .01, 72 h) could markedly reduce the ICH‐induced increase in TUNEL^+^ cells. However, the effects of PUE at 50 mg/kg and 100 mg/kg had no statistical difference (*P* > .05, 24 h and 72 h) (Figure 2C). Typically, macroscopical images of hematoma are shown (Figure 3A). PUE could significantly reduce the hematoma volume at 50 mg/kg (*P* < .05, 24 h; *P* < .001, 72 h) (Figure 3B). Besides, PUE (100 mg/kg) also could produce similar effects (*P* < .05, 24 h; *P* < .001, 72 h) after ICH (Figure 3B). Consistently, these beneficial effects of PUE at 50 mg/kg and 100 mg/kg on the hematoma size were no significant statistical difference (*P* > .05, 24 h and 72 h) (Figure 3B).

### 3.4 PUE could restrain ICH‐induced stimulation of NF‐κB pathway

**FIGURE 4:**
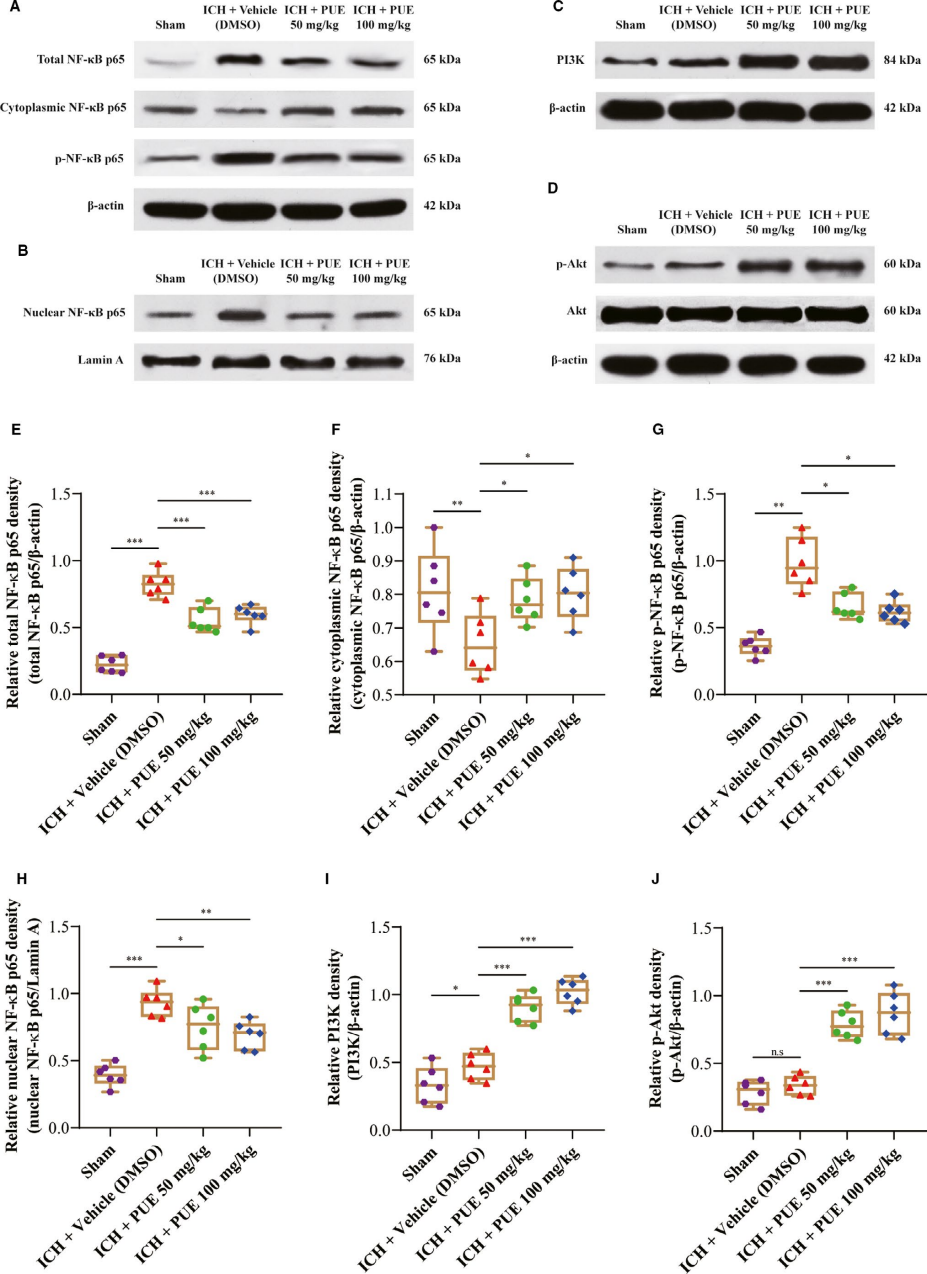
Effects of PUE delivery (50 and 100 mg/kg) on the expression levels of NF‐κB pathway and PI3K/Akt signal‐related proteins at 24 h post‐ICH. Typical WB protein bands of NF‐κB p65 (total, cytoplasmic, phosphorylated, and nuclear) are shown (A, B). Relatively quantitative analyses of total NF‐κB p65 (E), cytoplasmic NF‐κB p65 (F), p‐NF‐κB p65 (G), and nuclear NF‐κB p65 (H) protein levels were, respectively, exhibited (n = 6 rats/group; total NF‐κB p65, cytoplasmic NF‐κB p65, and nuclear NF‐κB p65: LSD test; p‐NF‐κB p65: Dunnett's T3 test). Similarly, typical WB protein bands of PI3K, p‐Akt, and Akt (C, D) were exhibited, and corresponding quantitative evaluations of PI3K (I), p‐Akt (J), and Akt (Figure 5S) (n = 6 rats/group; PI3K, p‐Akt, and Akt: LSD test) are shown. Values are indicated by means ± SD. ***P < .001; **P < .01; *P < .05; n.s: no statistical significance

**FIGURE 5:**
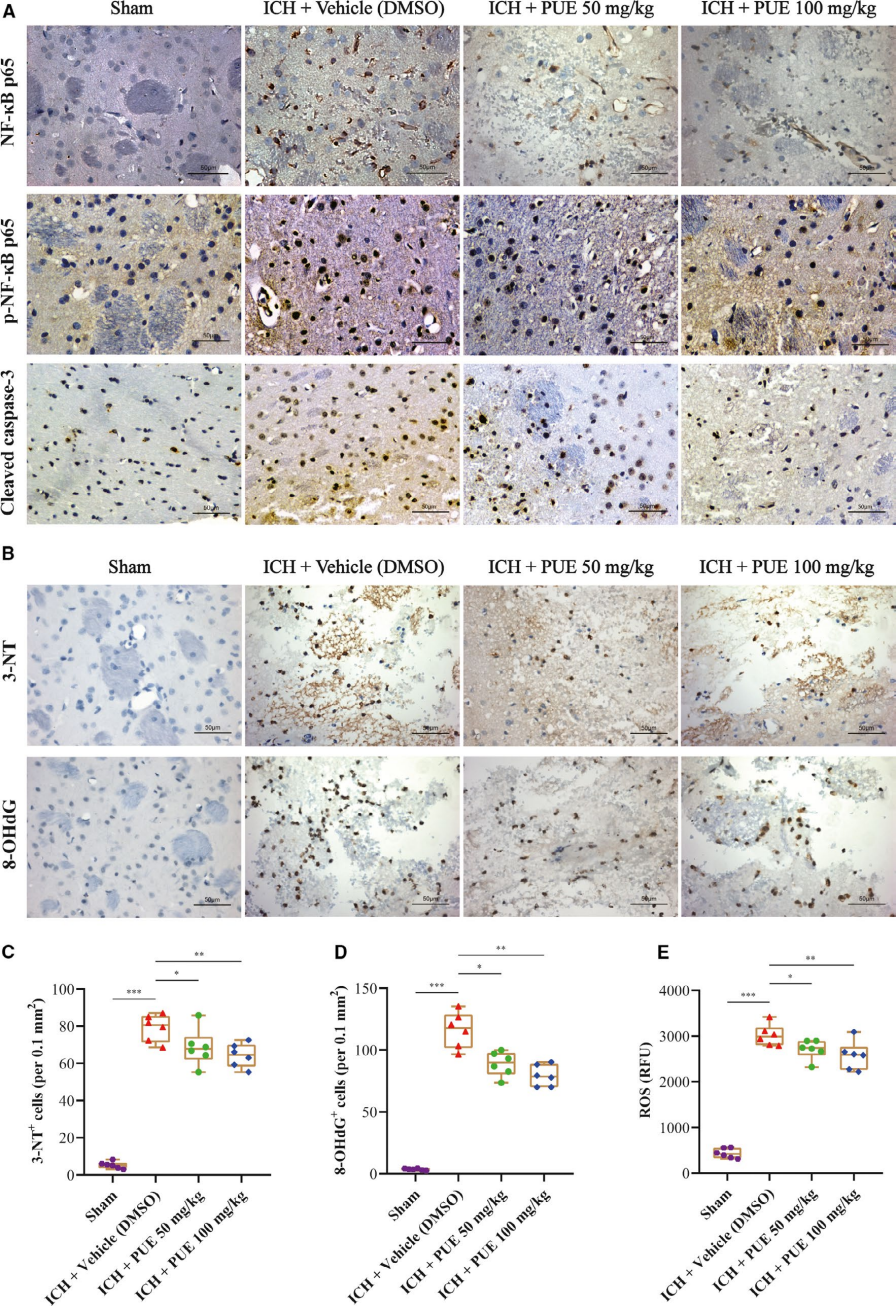
Effects of PUE (50 and 100 mg/kg) on the protein levels of NF‐κB p65, p‐NF‐κB p65, cleaved caspase‐3, and OS markers, and ROS content. At 24 h following ICH, typical IHC images of NF‐κB p65, p‐NF‐κB p65, and cleaved caspase‐3 proteins are shown (A) (n = 6 rats/group). Representative IHC images of OS markers 3‐NT and 8‐OHdG (B) and corresponding quantitative analyses (C, D) at 24 h post‐ICH (n = 6 rats/group; 3‐NT: LSD test; 8‐OHdG: Dunnett's T3 test). The ROS level of perihematomal brain tissue at 24 h after ICH was also quantitatively evaluated (E) (n = 6 rats/group; ROS: LSD test). Scale bar = 50 μm. Values are reported as means ± SD. ***P < .001; **P < .01; *P < .05

Research studies have suggested that PUE can suppress the activation of the NF‐κB signal pathway.^58^, ^59^, ^60^, ^61^ Our results also indicated that PUE (50 mg/kg) could markedly curb the upregulation of total NF‐κB p65 (*P* < .001), p‐NF‐κB p65 (*P* < .05), and nuclear NF‐κB p65 (*P* < .05) (Figure 4A,B,E,G,H), and increase the cytoplasmic NF‐κB p65 level (*P* < .05) (Figure 4A,F) at 24 h after ICH. Representative IHC images of NF‐κB p65 and p‐NF‐κB p65 were obtained at 24 h following ICH and are shown (Figure 5A). Similarly, PUE (100 mg/kg) also could markedly decrease the protein levels of total NF‐κB p65 (*P* < .001) (Figure 4A,E), p‐NF‐κB p65 (*P* < .05) (Figure 4A,G), and nuclear NF‐κB p65 (*P* < .01) (Figure 4B,H) and significantly raise the cytoplasmic NF‐κB p65 level (*P* < .05) (Figure 4A,F).

### 3.5 PUE could trigger PI3K/Akt signal activation after ICH

Moreover, the acquired results also show that PUE (50 mg/kg) could significantly upregulate the expression levels of PI3K (*P* < .001) (Figure 4C,I) and p‐Akt (*P* < .001) at 24 h after ICH (Figure 4D,J). Similarly, PUE (100 mg/kg) could obviously increase the protein levels of PI3K (*P* < .001) (Figure 4C,I) and p‐Akt (*P* < .001) at 24 h following ICH as well (Figure 4D,J). However, both two doses of PUE failed to alter the expression level of Akt (*P* > .05) (Figure 4D, Figure 5S).

### 3.6 PUE could restrain ICH‐induced activation of apoptosis signal

**FIGURE 6:**
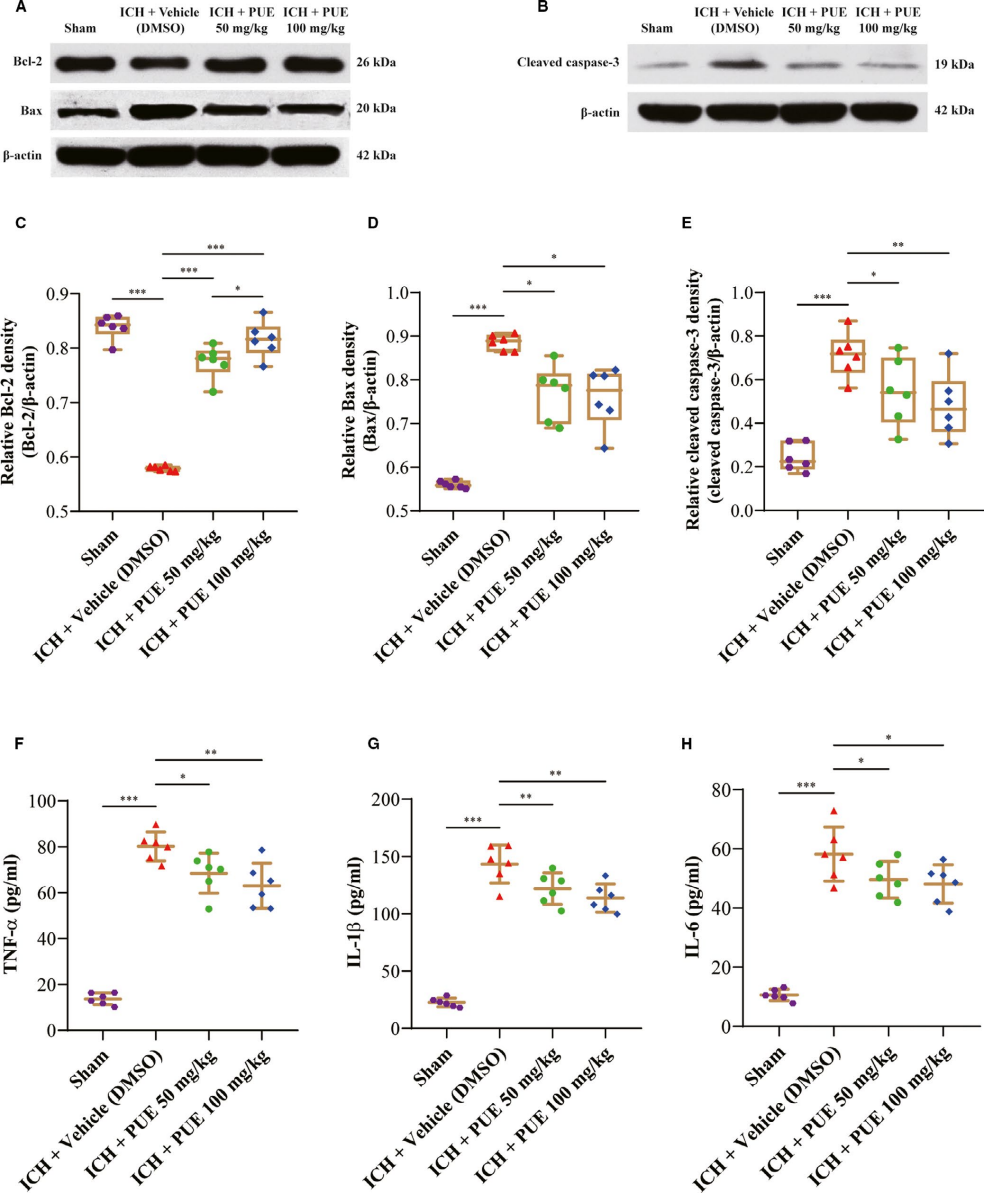
Effects of PUE delivery (50 and 100 mg/kg) on the expression levels of apoptosis‐related proteins and inflammatory cytokines at 24 h after ICH. Representative WB bands of Bcl‐2 and Bax (A), cleaved caspase‐3 (B) proteins and related quantitative analyses of Bcl‐2 (C), Bax (D), cleaved caspase‐3 (E) levels (n = 6 rats/group; Bcl‐2, and cleaved caspase‐3: LSD test; Bax: Dunnett's T3 test). The effects of PUE treatment on the levels of pro‐inflammatory cytokines TNF‐α, IL‐1β, and IL‐6 and correlatively quantitative analyses of TNF‐α (F), IL‐1β (G), and IL‐6 (H) (n = 6 rats/group; TNF‐α, IL‐1β, and IL‐6L: LSD test). Values are reported as means ± SD. ***P < .001; **P < .01; *P < .05

Typical WB bands of apoptosis signal‐related proteins (Bcl‐2, Bax, and cleaved caspase‐3) are shown (Figure 6A,B). The results showed that PUE at dosages of 50 mg/kg (*P* <.001) and 100 mg/kg (*P* < .001) could significantly increase the expression level of Bcl‐2 at 24 h after ICH (Figure 6C). Moreover, obtained results also suggested that PUE could notably compromise the upregulation of Bax (50 mg/kg and 100 mg/kg: *P* < .05) and that of cleaved caspase‐3 (50 mg/kg: *P* < .05; 100 mg/kg: *P* < .01) at 24 h following ICH (Figure 6D,E). Typical IHC images of cleaved caspase‐3 were acquired at 24 h after ICH and are shown (Figure 5A).

### 3.7 PUE could reduce OS injury markers and level of ROS after ICH

The massive production of ROS can cause severe OS, and uncontrolled OS is capable of hugely contributing to ICH‐induced EBI,^2^, ^5^, ^6^, ^62^ and PUE can alleviate OS‐induced injury by scavenging ROS.^7^, ^8^ Therefore, we propose that PUE could drop the levels of OS injury markers (8‐OHdG and 3‐NT) following ICH. Typical IHC images of perihematomal 3‐NT^+^ and 8‐OHdG^+^ cells are shown (Figure 5B). The results suggested that PUE (50 mg/kg and 100 mg/kg) could significantly downregulate the levels of perihematomal 3‐NT^+^ cells (50 mg/kg: *P* < .05; 100 mg/kg: *P* < .01) (Figure 5C) and 8‐OHdG^+^ cells (50 mg/kg: *P* <.05; 100 mg/kg: *P* <.01) (Figure 5D). Moreover, our results also indicated that at 24 h after ICH, the level of ROS notably increased (*P* < .001) (Figure 5E), and PUE treatment could effectively decrease perihematomal ROS production at 50 mg/kg (*P* < .05) (Figure 5E) and 100 mg/kg (*P* < .01) (Figure 5E), yet there was no obvious discrimination between two dosages (*P* > .05) (Figure 5E).

### 3.8 PUE could downregulate the levels of pro‐inflammatory cytokines after ICH

We performed ELISA to further investigate the role of PUE on inflammatory cytokine levels at 24 h after ICH. The results showed that after ICH, levels of inflammatory cytokines (TNF‐α, IL‐1β, and IL‐6) notably increased (*P* < .001) (Figure 6F,G,H), and PUE (50 mg/kg) could markedly drop the levels of TNF‐α (*P* < .05) (Figure 6F), IL‐1β (*P* <.01) (Figure 6G), and IL‐6 (*P* < .05) (Figure 6H). Similarly, the levels of TNF‐α (*P* < .01) (Figure 6F), IL‐1β (*P* < .01) (Figure 6G), and IL‐6 (*P* < .05) (Figure 6H) were all significantly lowered by PUE treatment at 100 mg/kg. However, the beneficial effects of two doses were similar (*P* >.05) (Figure 6F,G,H).

### 3.9 LY294002 could compromise PUE‐mediated beneficial effects after ICH

**FIGURE 7:**
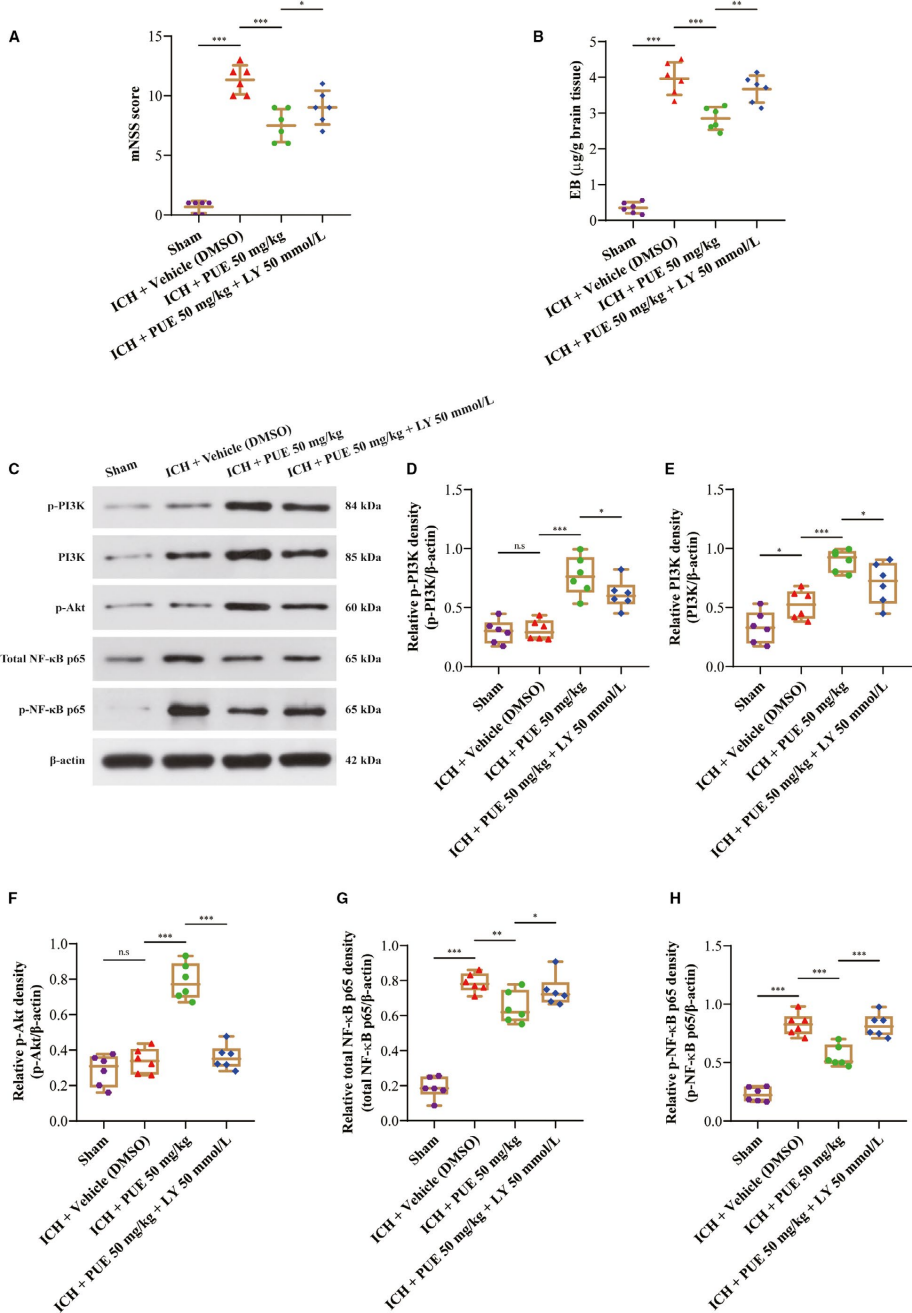
Effects of LY294002 injection on PUE‐mediated neuroprotection at 24 h following ICH. The mNSS score of neurological deficiency at 24 h after ICH is shown (A) (n = 6 rats/group; mNSS: LSD test). Assessment of BBB disruption with EB dyes (n = 6 rats/group; EB: LSD test) (B) was exhibited. The effects of LY294002 injection on the protein levels of p‐PI3K, PI3K, p‐Akt, total NF‐κB p65, and p‐NF‐κB p65 are shown by typical WB protein bands (C), and correspondingly, quantitative analyses of p‐PI3K (D), PI3K (E), p‐Akt (F), total NF‐κB p65 (G), and p‐NF‐κB p65 (H) levels were performed (n = 6 rats/group; p‐PI3K, PI3K, p‐Akt, total NF‐κB p65, and p‐NF‐κB p65: LSD test). Data are indicated by means ± SD. ***P < .001; **P < .01; *P < .05; n.s: no statistical significance

LY294002 (a specific inhibitor of the PI3K/Akt signal) was used to ulteriorly explore the protective mechanisms of PUE in ICH‐induced EBI, and the acquired results indicated that LY294002 could markedly weaken the beneficial effects of PUE on ICH‐induced behavioral deficiency indicated by increasing the mNSS scores (*P* < .05) (Figure 7A) and BBB disruption indexed by aggravating EB dye extravasation (*P* < .01) (Figure 7B) at 24 h after ICH.

### 3.10 LY294002 could recede PUE‐induced activation of PI3K/Akt signal and blockage of NF‐κB pathway at 24 h post‐ICH

Furthermore, LY294002 treatment could significantly downregulate the expression levels of p‐PI3K (*P* < .05) (Figure 7C,D), PI3K (*P* < .05) (Figure 7C,E), and p‐Akt (*P* < .001) (Figure 7C,F) proteins at 24 h following ICH. Besides, LY294002 was also shown to visibly enhance the expression levels of total NF‐κB p65 (*P* < .05) (Figure 7C,G) and p‐NF‐κB p65 (*P* < .001) (Figure 7C,H) at 24 h following ICH.

## 4 DISCUSSION

Increasing evidence has shown that OS and neuroinflammation are incredibly crucial for ICH‐induced EBI, which is characterized by massive brain cell apoptosis.^1^, ^2^, ^5^, ^6^, ^62^ Here, we found that PUE could efficiently alleviate ICH‐induced EBI by reducing the mNSS scores, brain cell apoptosis, and hematoma volume and by improving the histological injury, BBB disruption, and brain edema. Moreover, we also found that PUE could notably repress NF‐κB pathway activation and promote PI3K/Akt signal stimulation by upregulation of cytoplasmic NF‐κB p65, PI3K, and p‐Akt and downregulation of total, phosphorylated, and nuclear NF‐κB p65. Besides, PUE could also significantly inhibit ICH‐induced OS and the production of pro‐inflammatory cytokines. Finally, we ulteriorly found that LY294002 could notably compromise PUE’s brain beneficial effects by aggravating behavioral deficiency and BBB disruption, upregulating total and p‐NF‐κB p65 levels, downregulating PI3K, p‐PI3K, and p‐Akt levels after ICH. This was the first time to explore in detail the neuroprotective effects of PUE on ICH‐induced EBI and related molecular mechanisms. The potential molecular mechanisms of PUE’s brain‐protective effects are shown (Fig. S6).

Research studies have confirmed that activation of NFthe ‐κB signal pathway widely exists in various kinds of CNS damages, including ischemic stroke,^63^, ^64^, ^65^ ICH,^44^, ^45^, ^66^ and SAH,^36^, ^67^ and its repression could notably reduce these brain impairments.^36^, ^63^, ^64^, ^65^, ^67^ In our previous research, we also have verified that NF‐κB signal pathway‐related proteins were significantly upregulated after ICH, and its suppression could markedly alleviate ICH‐induced EBI and neurobehavioral deficiency.^44^, ^45^ Similarly, our current results suggest that PUE could significantly improve ICH‐induced EBI and related molecular mechanisms possibly by PI3K/Akt signal activation‐mediated suppression of the NF‐κB pathway.

Stimulation of the PI3K/Akt signal has also been reported by different groups that it could notably mitigate acute CNS disorder‐induced brain injury, including ICH ^40^, ^41^, ^42^, ^43^ and SAH.^48^, ^49^, ^68^, ^69^, ^70^ An α7‐nicotinic acetylcholine receptor agonist PHA‐543613 can significantly improve ICH‐induced brain injury and improve neurological deficiency by PI3K/Akt signal activation‐mediated repression of glycogen synthase kinase‐3β and β‐catenin stabilization, and wortmannin (a specific inhibitor of PI3K/Akt signal) can notably weaken these brain‐protective effects.^40^, ^43^ Similarly, posttreatment of [Gly14]‐humanin and transplantation of bone marrow mesenchymal stem cells also can generate neuroprotective effects by triggering the PI3K/Akt signal.^41^

PUE [8‐(β‐D‐glucopyranosyl)‐7‐hydroxy‐3‐(4‐hydroxyphenyl)‐4H‐1‐benzopyran‐4‐one, C_21_H_20_O_9_], chemical structure (Figure 1A), belongs to a bioactive isoflavone compound extracted from the roots of *Pueraria lobata*.^7^, ^8^, ^71^, ^72^, ^73^ Research studies have shown that PUE holds intensively neuroprotective effects for various kinds of CNS disorders, including stroke, SAH, TBI, PD, AD, and SCI, through multiple activities, such as antioxidation, anti‐inflammation, and antiapoptosis.^7^, ^8^, ^17^, ^18^, ^19^, ^20^, ^21^, ^22^, ^23^, ^24^, ^26^, ^27^, ^28^, ^29^, ^30^, ^31^, ^32^, ^33^ PUE can exert brain‐protective effects on ischemia/reperfusion‐mediated brain injury by the suppression of apoptosis/necrosis, autophagy activation, and/or inflammatory response ^9^, ^10^, ^11^, ^12^, ^13^, ^14^, ^15^ by the promotion of X‐chromosome‐linked inhibitor of apoptosis protein,^15^ erythropoietin activity,^13^ stimulation of Janus‐activated kinase‐2 and signal transducers and activators of transcription‐3,^12^ inhibition of caspase‐3 activity and expression,^15^ NF‐κB signal pathway,^11^, ^12^ Toll‐like receptor‐4, myeloid differentiation factor‐88,^11^ and autophagy level.^9^ Besides, PUE could also reduce hypobaric hypoxia‐mediated acute lung and cerebrum injury,^74^ nickel‐induced liver injury,^75^ and diabetes and chronic constriction injury‐induced neuropathic pain by suppressing the activation of the NF‐κB pathway.^23^ Moreover, PUE could effectively reduce SAH‐induced neurological deficiency and EBI by restraining the Bax/cleaved caspase‐3‐induced apoptotic signal pathway and stimulating the sirtuin‐3/superoxide dismutase‐2‐mediated anti‐apoptotic signal as well.^17^

Puerarin is also well‐confirmed to produce various beneficial effects by activation of the PI3K/Akt signal pathway. A recent report has exhibited that PUE could ameliorate OS‐induced neurodegeneration by stimulating the PI3K/Akt signal after TBI, and the specific inhibitor of the PI3K/Akt signaling pathway LY294002 could markedly reduce PUE’s protective effects.^18^ We also found that PUE could exert similar neuroprotective effects, including activation of the PI3K/Akt signal, inhibition of brain cell apoptosis, and improvement of brain injury, and LY294002 could efficaciously compromise PUE’s brain‐protective effects. Moreover, our results further suggested that stimulation of the PI3K/Akt signal could reduce ROS formation and neuroinflammatory response induced by NF‐κB pathway activation in ICH‐induced EBI. Besides, PUE is also reported to improve AD‐induced cognitive dysfunction, OS and neuronal apoptosis,^26^, ^27^, ^72^, ^76^ and PD‐induced behavioral deficits and depletion of dopamine due to loss of dopaminergic neurons ^29^ by activating the PI3K/Akt signal.^29^, ^72^, ^76^

Research studies have suggested that the PI3K/Akt signal is the powerful upstream regulator of the NF‐κB pathway.^36^, ^67^, ^77^ Stimulation of the PI3K/Akt signal could dramatically lower the p‐NF‐κB p65 level and release of inflammatory cytokines (TNF‐α and IL‐1β), and LY294002 could reverse these effects. Hence, neuroinflammation, neutrophil infiltration, microglial activation, and BBB disruption were decreased, and neurological deficits were improved after SAH.^36^, ^67^, ^77^ This was also consistent with our results that PUE‐mediated PI3K/Akt signal activation could relieve NF‐κB pathway stimulation‐induced brain‐detrimental effects.

Some potential limitations deserve special attention in our study. First, PUE can generate multiple beneficial effects by manipulation of different signal pathways, but we primarily focused on PI3K/Akt signal activation‐mediated suppression of the NF‐κB pathway. Hence, PUE may exert neuroprotective effects by other signal pathways such as the Nrf2 signaling pathway ^32^ and iron metabolism pathway.^25^ Second, the collagenase‐induced rat ICH model itself cannot completely mirror the pathophysiological process of ICH patients. Thus, results from our research might need to be further verified with other ICH models such as autologous blood and hemoglobin injection and be carefully explained.

Overall, our results have indicated that PUE could notably improve ICH‐induced EBI and neurological deficiency, and related mechanisms might be involved in the suppression of NF‐κB signal pathway activation‐induced brain injury partly by the triggering of PI3K/Akt signal pathway‐mediated neuroprotection. Our findings might provide a promising therapeutic selection for ICH‐induced EBI.

## CONFLICTS OF INTEREST

All authors listed in this manuscript declared that no any conflicts of interest existed.

## AUTHOR CONTRIBUTION

**Jun Zeng:** Conceptualization (equal); Data curation (equal); Formal analysis (equal); Investigation (equal); Methodology (equal); Software (equal); Validation (equal); Visualization (equal); Writing‐original draft (equal). **Shizhong Zheng:** Conceptualization (equal); Data curation (equal); Formal analysis (equal); Investigation (equal); Methodology (equal); Software (equal); Validation (equal); Visualization (equal); Writing‐original draft (equal). **Yizhao Chen:** Conceptualization (equal); Data curation (equal); Funding acquisition (lead); Project administration (lead); Resources (lead); Supervision (lead); Writing‐review & editing (lead). **Yaoming Qu:** Formal analysis (supporting); Investigation (supporting); Methodology (supporting); Software (supporting); Visualization (supporting); Writing‐review & editing (supporting). **Jiayu Xie:** Formal analysis (supporting); Investigation (supporting); Methodology (supporting); Software (supporting); Visualization (supporting); Writing‐review & editing (supporting). **Enhui Hong:** Formal analysis (supporting); Investigation (supporting); Methodology (supporting); Software (supporting); Visualization (supporting); Writing‐review & editing (supporting). **Hongzhu Lv:** Formal analysis (supporting); Investigation (supporting); Methodology (supporting); Software (supporting); Visualization (supporting); Writing‐review & editing (supporting). **Rui Ding:** Formal analysis (supporting); Investigation (supporting); Methodology (supporting); Software (supporting); Visualization (supporting); Writing‐review & editing (supporting). **Liang Feng:** Formal analysis (supporting); Investigation (supporting); Methodology (supporting); Software (supporting); Visualization (supporting); Writing‐review & editing (supporting). **Zhichong Xie:** Formal analysis (supporting); Investigation (supporting); Methodology (supporting); Software (supporting); Visualization (supporting); Writing‐review & editing (supporting).

## Supporting information

Fig. S1.

Fig. S2.

Fig. S3.

Fig. S4.

Fig. S5.

Fig. S6.

Supplementary Material

## Data Availability

Contact the corresponding author for data requests.
