# Supplementary figures and images for: Puerarin attenuates intracerebral hemorrhage‐induced early brain injury possibly by PI3K/Akt signal activation‐mediated suppression of NF‐κB pathway
Source: J Cell Mol Med. 2021 Jun 27;25(16):7809–24. doi: 10.1111/jcmm.16679 (PMC8358853; doi:10.1111/jcmm.16679)

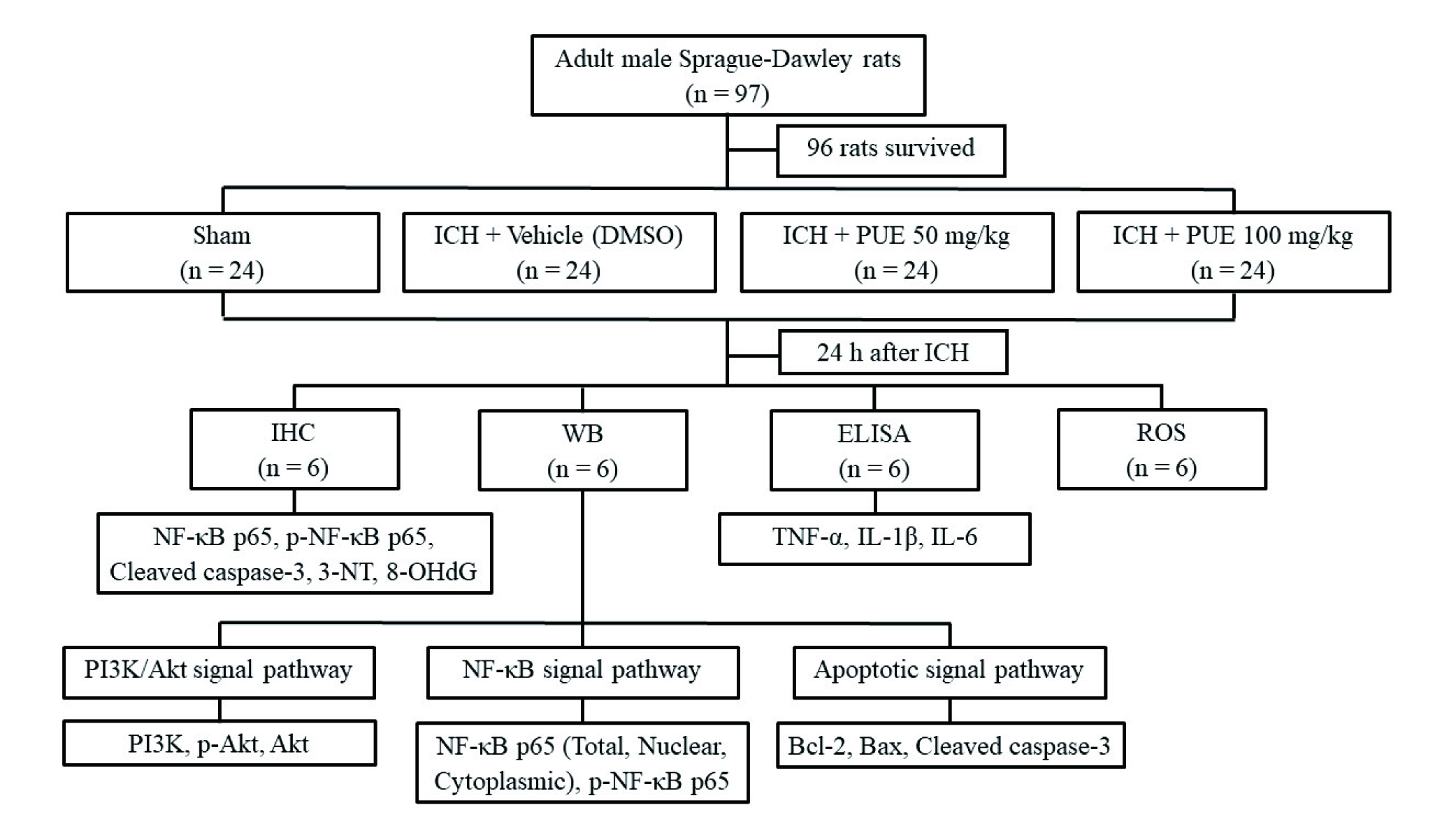

Supplement: Supplementary file 2 — Fig. S2. [file JCMM-25-7809-s001.tif]

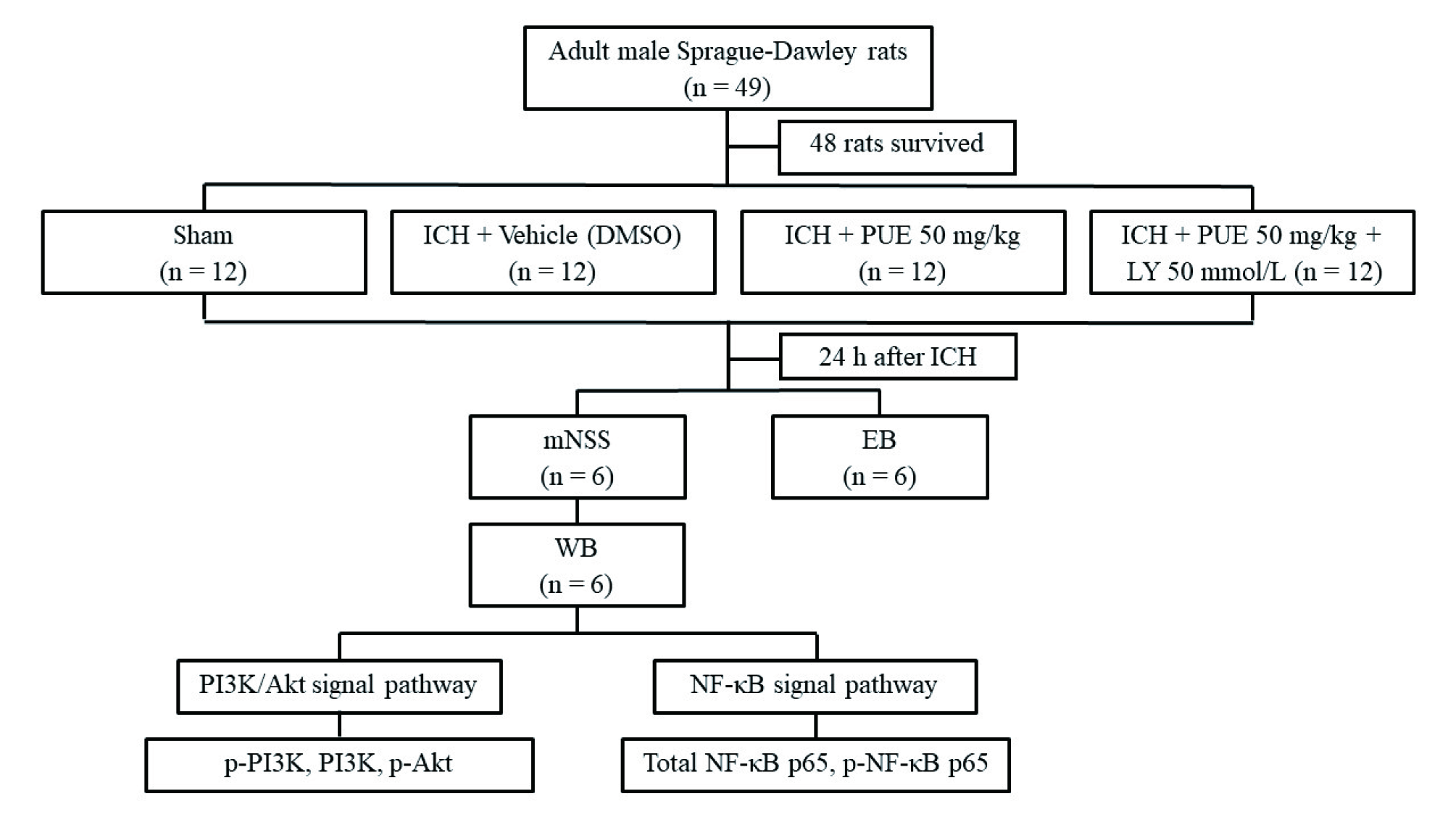

Supplement: Supplementary file 3 — Fig. S3. [file JCMM-25-7809-s003.tif]

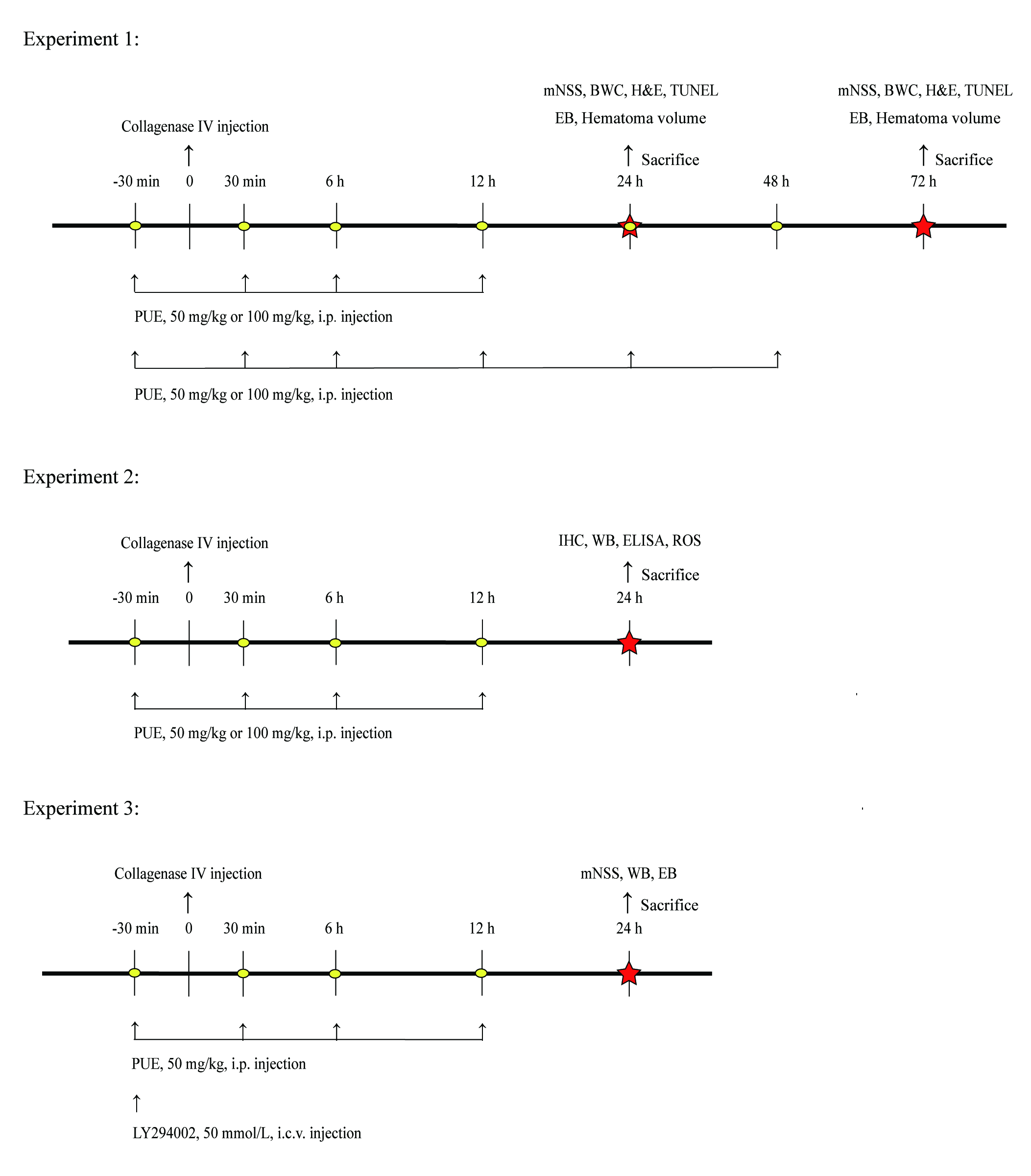

Supplement: Supplementary file 4 — Fig. S4. [file JCMM-25-7809-s005.tif]

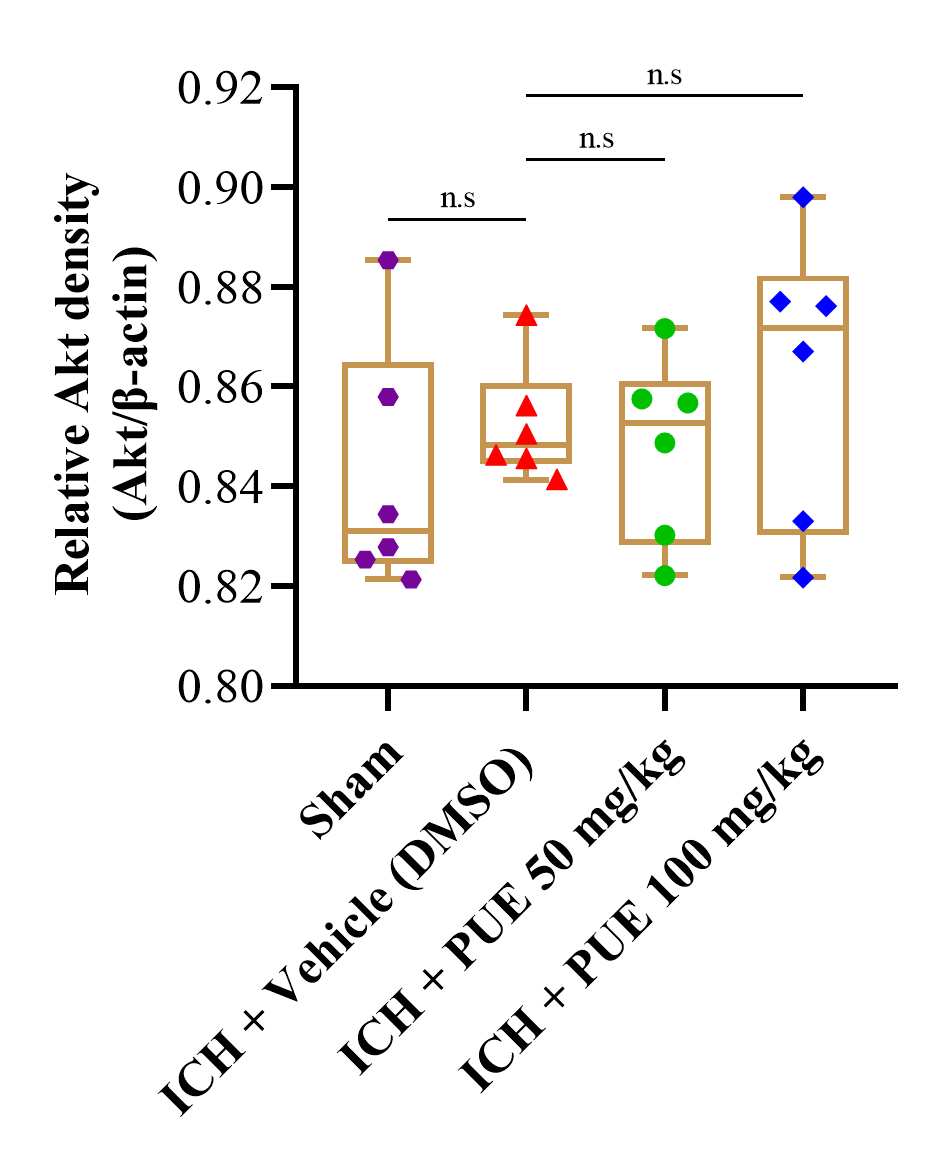

Supplement: Supplementary file 5 — Fig. S5. [file JCMM-25-7809-s006.tif]

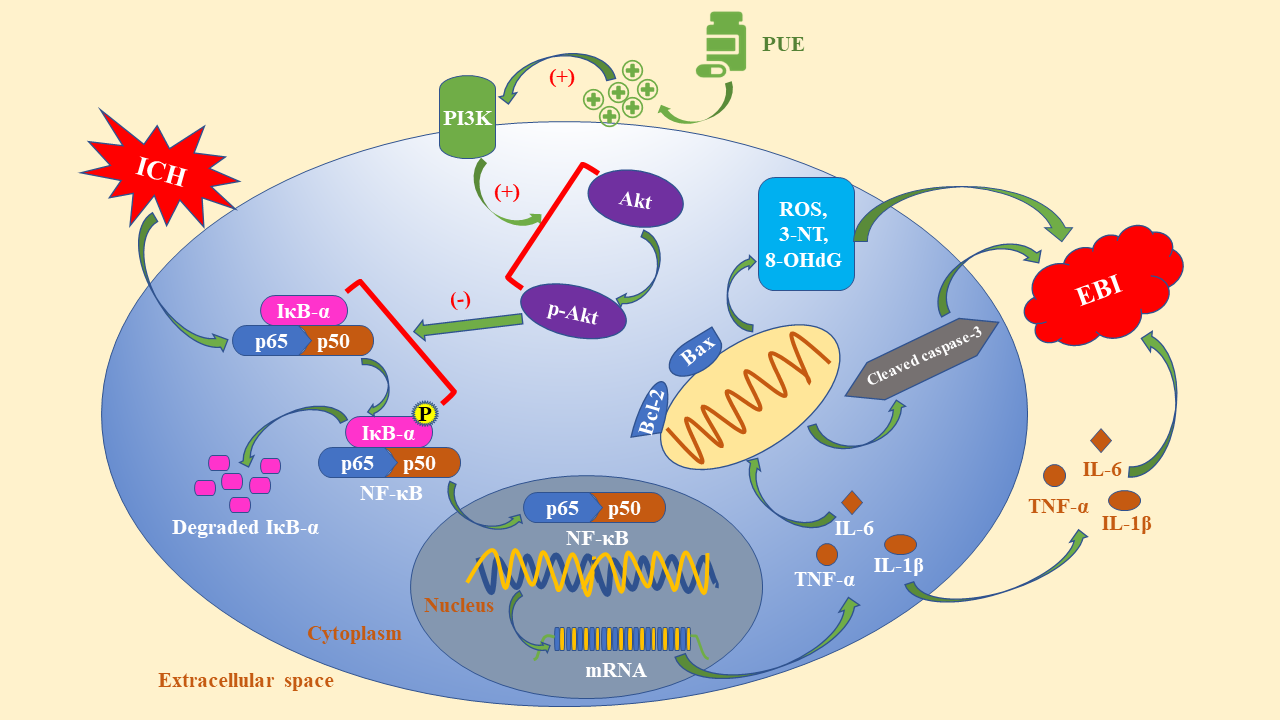

Supplement: Supplementary file 6 — Fig. S6. [file JCMM-25-7809-s007.tif]
