# Supplementary material for: Puerarin attenuates intracerebral hemorrhage‐induced early brain injury possibly by PI3K/Akt signal activation‐mediated suppression of NF‐κB pathway
Source: J Cell Mol Med. 2021 Jun 27;25(16):7809–24. doi: 10.1111/jcmm.16679 (PMC8358853; doi:10.1111/jcmm.16679)
Supplement: Supplementary file 7 — Supplementary Material [file JCMM-25-7809-s004.doc]

**1. Figure legends**

**Figure. 1S. The effects of PUE treatment on EBI after ICH**

Rats were equally and randomly divided into four groups [48 rats/group: sham, ICH + Vehicle (DMSO), ICH + PUE 50 mg/kg and ICH + PUE 100 mg/kg]. Rats were scored with an mNSS scale (n = 12 rats/group) at 24 and 72 h after ICH, except that the rats to conduct measurement of EB dye extravasation (n = 6 rats/group) and evaluation of hematoma volume (n = 6 rats/group). Then, rats were dedicated to perform H&E staining (n = 6 rats/group), BWC evaluation (n = 6 rats/group) and TUNEL staining (n = 6 rats/group).

**Figure. 2S. Potential molecular mechanisms of PUE’ neuroprotective effects on ICH-induced EBI**

Rats were randomized and equalized into four groups [24 rats/group: sham, ICH + Vehicle (DMSO), ICH + PUE 50 mg/kg and ICH + PUE 100 mg/kg]. All rats were dedicated to execute WB (n = 6 rats/group), IHC staining (n = 6 rats/group), ELISA (n = 6 rats/group) and ROS detection (n = 6 rats/group) at 24 h after ICH.

**Figure. 3S. The effects of PI3K/Akt signal pathway inhibitor LY294002 on PUE-mediated neuroprotection after ICH**

We randomly and evenly divided rats into four groups [12 rats/group: sham, ICH + Vehicle (DMSO), ICH + PUE 50 mg/kg and ICH + PUE 50 mg/kg + LY294002 (LY) 50 mmol/L]. After evaluated with mNSS scale at 24 h after ICH (n = 6 rats/group), rats were dedicated to conduct WB (n = 6 rats/group). EB dye extravasation detection was also performed in another group of rats (n = 6 rats/group).

**Figure. 4S.** **The schedule of research**

During our research, the generation of rat ICH model, treatments of different agents, and corresponding experimental assessments in given time points were detailedly shown by the schedule.

**Figure. 5S. The effects of PUE on the expression level of Akt protein after ICH**

Quantitative analysis of the WB band for Akt protein at 24 h after ICH induction (n = 6 rats/group). ***: *p* < 0.001; **: *p* < 0.01; *: *p* < 0.05; n.s: no statistical significance.

**Figure. 6S. Schematic diagram of potential molecular mechanisms**

The potential molecular mechanisms of PUE's neuroprotective effects on EBI after ICH were shown. Research results have indicated that PUE could significantly reduce ICH-induced EBI and neurological deficits, and relatively underlying mechanisms might be involved in PI3K/Akt signal activation-mediated suppression of the NF-κB pathway.

**2. Abbreviations**

ICH: intracerebral hemorrhage; OS: oxidative stress; EBI: early brain injury; PUE: puerarin; CNS: central nervous system; SAH: subarachnoid hemorrhage; TBI: traumatic brain injury; SCI: spinal cord injury; AD: Alzheimer's disease; PD: Parkinson's disease; PI3K: phosphatidylinositol 3-kinase; NF-κB: nuclear factor-κB; TNF-α: tumor necrosis factor-α; IL-6: interleukin-6; IL-1β: interleukin-1β; DMSO: dimethylsulfoxide; PBS: phosphate-buffered saline; mNSS: modified neurological severity score; H&E: hematoxylin & eosin; IHC: immunohistochemistry; TUNEL: terminal deoxynucleotidyl transferase-mediated biotinylated-dUTP nick-end labeling; BWC: brain water content; BBB: blood-brain barrier; EB: Evans blue; CST: Cell Signaling Technology; 3-NT: 3-nitrotyrosine; 8-OHdG: 8-hydroxy-2'-deoxyguanosine; WB: western blot; ELISA: enzyme-linked immunosorbent assay; ROS: reactive oxygen species; DCFH-DA: 2, 7-dichlorofluorescein diacetate; SD: standard deviation; ANOVA: analysis of variance; LSD: least significant difference.
